# COVID-19 pandemic: CT chest in COVID-19 infection and prediction of patient’s ICU needs

**DOI:** 10.1186/s43055-021-00515-z

**Published:** 2021-05-26

**Authors:** Ahmed M. Osman, Ahmed M. Abdrabou, Reham M. Hashim, Faisal Khosa, Aya Yasin

**Affiliations:** 1 Radiology Department, Faculty of Medicine, Ain Shams University, Cairo, Egypt; 2 Anesthesia, ICU, and Pain Management Department, Faculty of Medicine, Ain Shams University, Cairo, Egypt; 3 Radiology Department, Vancouver General Hospital, University of British Columbia, Vancouver, Canada

**Keywords:** Coronavirus, Computed tomography (CT), Ground-glass opacity (GGO), Pandemic, High-resolution CT

## Abstract

**Background:**

With the tremendous rise in COVID-19 infection and the shortage of real-time reverse transcription-polymerase chain reaction (RT-PCR) testing, we aimed to assess the role of CT in the detection of COVID-19 infection and the correlation with the patients’ management. A retrospective study was conducted on 600 patients who presented with symptoms suspicious for COVID-19 infection between March and the end of June 2020. The current study followed the RSNA recommendations in CT reporting and correlated with the RT-PCR. CT was reviewed and the severity score was correlated with the patient’s management.

**Results:**

Four hundred sixty-six patients were included with a mean age of 46 + 14.8 years and 63.3 % were males. Three hundred forty patients were confirmed positive by RT-PCR. CT sensitivity was 92.6% while the RT-PCR was the reference. The CT specificity showed a gradual increase with the CT probability reaching 97.6% with high probability CT features. Ground-glass opacities (GGO) was the commonest findings 85.9% with a high incidence of bilateral, peripheral, and multilobar involvement (88%, 92.8%, and 92.8% respectively). Consolidation was found in 81.5% of the ICU patients and was the dominant feature in 66.7% of the ICU cases. CT severity score was significantly higher in ICU patients with a score of ≥ 14.

**Conclusions:**

COVID-19 infection showed typical CT features which can be used as a rapid and sensitive investigation. Two CT phenotypes identified with the predominant consolidation phenotype as well as severity score can be used to determine infection severity and ICU need.

## Backgrounds

Coronaviruses belong to the Coronaviridae family. Coronaviruses include six well-known strains, two of which cause severe respiratory distress including severe acute respiratory syndrome (SARS) and the Middle East respiratory distress syndrome (MERS) [1].

In December 2019, a new strain of coronaviruses was described in Wuhan, China, and was called the novel coronavirus 2019 (nCoV-19) then by February, WHO gave the official denomination as COVID-19. It causes severe pneumonia, called novel COVID-19 infection pneumonia (NCIP) which has also been termed severe acute respiratory syndrome coronavirus 2 (SARS CoV2). Novel COVID-19 strain is transmitted from human to human and causing a wide variety of symptoms [2–4]. It uses angiotensin-converting enzyme 2 (ACE2) receptors in the respiratory tract, causing pulmonary interstitial damage followed by parenchymal changes that vary according to the stage and the severity of infection [5].

Novel COVID-19 virus has changed the world with the unusual use of masks, gloves, and sanitizers. It has also caused prolonged school closures, job losses, and economic hardship. The World Health Organization (WHO) announced an ongoing outbreak as a global public health emergency on January 30, 2020, which increased to a high alert on February 28, 2020. According to the WHO report on June 30, 2020, the number of infections reached over 10 million with about 503,000 total deaths [6]. In Egypt, the first case was diagnosed on February 14, 2020, then on June 30, 2020, the number of cases reached nearly 67,000 and 2900 deaths [6, 7].

The diagnosis of novel COVID-19 depends on real-time reverse transcription-polymerase chain reaction (RT-PCR) which is considered the gold standard for diagnosis, being highly specific. Yet, its limitation includes low sensitivity, high cost, and sometimes, early false-negative results [8]. The sensitivity of RT-PCR is 42-71%, while computed tomography (CT) sensitivity is 60-98% and specificity is 25-53% according to previous research [8]. RT-PCR may take a few days to turn from negative to positive in some patients [9].

Recent studies highlight the role of chest CT in the diagnosis of cases with novel COVID-19 infection, different CT features, CT types and phenotypes according to dominant features, CT severity, monitoring the progression, and assessment of the therapeutic response [8, 10–12].

This study aimed to assess the diagnostic accuracy of chest CT in the detection of COVID-19 infection, in comparison to the RT-PCR following the RSNA recommendations in CT interpretation showing the diagnostic accuracy of each probability of the RSNA recommendations. A secondary aim was to evaluate the different imaging features in positive cases highlighting the different CT phenotyping and the CT features reflecting the severity and finally, establish a relation between the chest CT severity score and the need for ICU admission.

## Methods

### Patients

This is a retrospective study conducted on 600 patients, suspected of novel COVID-19 infection between March 2020 and the end of June 2020 at a single tertiary institution. All patients underwent a high-resolution CT chest without intravenous contrast using dose reduction options available in our CT machine by using low kVp and low mAs as possible. The institutional ethical committee approved the study with a waiver of the written informed consent.

### Inclusion criteria

Any patient presented to the emergency department (ER) or outpatients clinics with clinical suspicious of COVID-19 infection (e.g., low-grade fever, dyspnea, cough, diarrhea, contact with known case with COVID-19 infection, and loss of smell or taste) who underwent non contrast chest CT and laboratory investigation to diagnose/exclude COVID-19 infection including RT-PCR.

### Exclusion criteria

Patients with unavailable RT-PCR or patients with no data about the management strategy. Also, patients with inadequate image interpretation due to respiratory motion artifacts and patients who were admitted to ICU due to medical conditions rather than COVID-19 infection.

### CT technique

- CT machine: 80-slice CT machine (Prime Aquilion, Toshiba, USA). The infection control parameters were applied under the guidance of the hospital infection control unit.
- No specific preparations were needed. The patients were scanned in a supine position with the arm above the head to avoid artifacts. Image acquisition was at 1.25 mm thickness, 0.625 mm interval using 512 × 512 matrix, tube speed 35 mm/rotation with 0.5 s rotation time. The kVp and mAs was used as low as possible controlled by the operator before scanning to get low radiation dose CT as possible.
- Image processing and interpretation: The images were transferred to the workstation for reviewing the axial slices along with multi-planar reformation. The image analysis was performed by two blinded radiologists experienced in chest imaging with at least 3 years of post-fellowship experience and any difference was resolved by consensus to avoid any inter-observer disagreement being not the scope of the current study. They were blinded to the aim of the study and the patients’ clinical symptoms. The following imaging findings were registered:
  - Presence or absence of ground-glass opacity (GGO): site (peripheral or central, unilateral or bilateral, and posterior distribution), shape (rounded, linear, patchy, or confluent). Also, if the lesions were uni-lobar or multi-lobar (i.e., affection of 2 lobes or more).
  - Presence or absence of consolidation: type (segmental, subsegmental, or lobar) and whether it was a predominant feature compared to the GGO.
  - Presence or absence of other features, e.g., crazy paving pattern, curvilinear lines, vascular dilatation sign, bronchiectasis, pleural effusion, lymphadenopathy, nodules, or cavitation.
  - Reporting whether the GGO or the consolidation was the dominant CT pattern in each case.
- Assessment of the CT probability: the CT probability for COVID-19 infection was expressed as high, intermediate, low probability, and negative which was followed according to the rules endorsed by the Radiological Society of North America (RSNA) to be equivalent to typical, indeterminate, atypical, and negative classification respectively [9].
- Assessment of the CT severity: In positive cases, the CT severity was retrospectively calculated. Each lung was divided into three zones: upper (above the carina), middle while lower (below the inferior pulmonary vein). Each zone was evaluated for the percentage of involvement with a score of 1 given if < 25% involvement and score 2 if between ≥ 25% and ≤ 50% involvement, score 3 if 50% and 75% involvement, and score 4 if 75% involvement and greater. The maximum score for the 6 zones was 24 [13, 14]. The CT severity score was compared with management decisions.

### RT-PCR assessment

Each patient had a nasopharyngeal or oropharyngeal swab which was repeated within 48 h intervals up to three times only if inadequate or negative yet with typical high probability chest CT criteria.

Data collection about the clinical decision taken at the time of presentation either home isolation, hospitalization, or the need for ICU admission which was taken by the triage team following the hospital guidelines for COVID-19 management depending on symptoms, laboratory findings, oxygen saturation, and presence of risk factors for disease progression [15].

### Sample size calculation

Using the PSAA-11 program and according to Simpson et al. [9] assuming sensitivity of CT for diagnosis of COVID-19 infection equal 70%, the proportion of patients with positive RT-PCR = 50%, a sample size of at least 100 patients can detect this sensitivity with power 80% and setting ά-error at 0.05.

### Statistical methods

The collected data were analyzed using IBM SPSS statistics (Statistical Package for Social Sciences) software version 22.0, IBM Corp., Chicago, USA, 2013. Descriptive statistics were done for quantitative data as minimum and maximum as well as mean ± SD (standard deviation) for quantitative normally distributed data, while it was done for qualitative data as number and percentage.

Inferential analysis was done for quantitative variables using the Shapiro-Wilk test for normality testing, independent *t* test in cases of two independent groups with normally distributed data, ANOVA test with post Bonferroni test for more than two independent groups with normally distributed data. In qualitative data, inferential analysis for independent variables was done using chi-square test for differences between proportions and Fisher’s exact test for variables with post hoc Bonferroni test. ROC curve was used to evaluate the performance of different tests and to differentiate between certain groups. The level of significance was taken as *P* value < 0.050 is significant.

## Results

### Demographic data

**Fig. 1:**
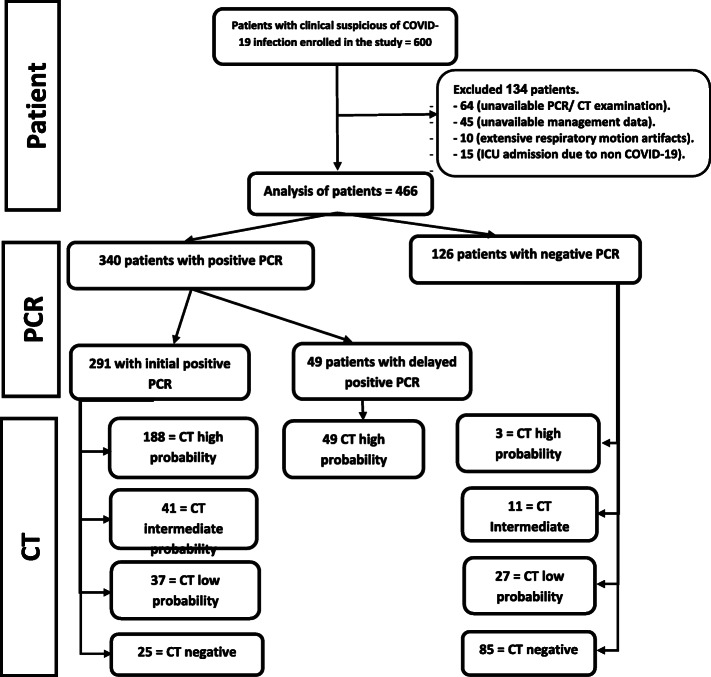
Flow chart of the patient under study

This is a retrospective cross-sectional study that was conducted on 466 patients, suspected clinically to have novel COVID-19 infection, after excluding 134 patients (Fig. 1). The mean age of the patients was 46 years + 14.8 years (ranged from 3 months to 84 years old). In the current study, 39 pediatric patients were found among the patient samples (age < 18 years), only 11 patients of them were positive for COVID-19 by RT-PCR. An overall 63.3% of the patients were males. The male to female ratio for the positive cases was 1.7 (Table 1). The range between the CT scanning and the onset of clinical symptoms was 2-7 days.

**Table 1:** Description of all cases and comparison according to RT-PCR result

Variables		Total (*N* = 466)	Positive (*N* = 340)	Negative (*N* = 126)	*P* value
**Mean age (years)**		46.0 ± 14.8	38.2 ± 21.2	44.0 ± 17.1	^< 0.001 *
**Sex**	**Male**	295 (63.3%)	212 (62.4%)	83 (65.9%)	# 0.484
	**Female**	171 (36.7%)	128 (37.6%)	43 (34.1%)	
**CT probability**	**High**	240 (51.5%)	237 (69.7%)	3 (2.4%)	# < 0.001*
	**Intermediate**	52 (11.2%)	41 (12.1%)	11 (8.7%)	
	**Low**	64 (13.7%)	37 (10.9%)	27 (21.4%)	
	**Negative**	110 (23.6%)	25 (7.4%)	85 (67.5%)	
**GGO (ground-glass opacity)**		306 (65.7%)	292 (85.9%)	14 (11.1%)	# < 0.001*
GGO rounded		169 (55.2%)	162 (55.5%)	7 (50.0%)	# 0.687
GGO linear		68 (22.2%)	65 (22.3%)	3 (21.4%)	§ 1.000
GGO patchy		52 (17.0%)	49 (16.8%)	3 (21.4%)	§ 0.714
GGO confluence		39 (12.7%)	39 (13.4%)	0 (0.0%)	§ 0.229
GGO diffuse		4 (1.3%)	3 (1.0%)	1 (7.1%)	§ 0.172
GGO laterality	Unilateral	37 (12.1%)	35 (12.0%)	2 (14.3%)	# 0.681
	Bilateral	269 (87.9%)	257 (88.0%)	12 (85.7%)	
GGO location	Peripheral	275 (89.9%)	271 (92.8%)	4 (28.6%)	# < 0.001*
	Central	31 (10.1%)	21 (7.2%)	10 (71.4%)	
GGO lobar affection	Multilobar	285 (93.1%)	271 (92.8%)	14 (100.0)	# 0.609
	Unilobar	21 (6.9%)	21 (7.2%)	0 (0.0%)	
**Crazy paving**		162 (34.8%)	154 (45.3%)	8 (6.3%)	# < 0.001*
**Vascular enlargement**		104 (22.3%)	100 (29.4%)	4 (3.2%)	# < 0.001*
**Consolidation**		128 (27.5%)	98 (28.8%)	30 (23.8%)	# 0.281
Consolidation type	Lobar	42 (32.8%)	25 (25.5%)	17 (56.7%)	# 0.001 *
	Segmental/subsegmental	86 (67.2%)	73 (74.5%)	13 (43.3%)	
**Consolidation > GG**		77 (16.5%)	52 (15.3%)	25 (19.8%)	# 0.240
**Curvilinear opacity**		94 (20.2%)	90 (26.5%)	4 (3.2%)	# < 0.001*
**Pleural effusion**		62 (13.3%)	38 (11.2%)	24 (19.0%)	# 0.026 *
**Fibrosis**		22 (4.7%)	17 (5.0%)	5 (4.0%)	0.641
**LN (lymphadenopathy)**		17 (3.6%)	11 (3.2%)	6 (4.8%)	§ 0.415
**Nodules**		23 (4.9%)	11 (3.2%)	12 (9.5%)	# 0.005 *
**Bronchiectasis**		10 (2.1%)	8 (2.4%)	2 (1.6%)	§ 1.000
**Cavitation**		6 (1.3%)	1 (0.3%)	5 (4.0%)	§ 0.006 *

^^^Independent t test

^#^Chi square test

^§^Fisher’s exact test

^*^Significant

### The performance of CT compared to the RT-PCR results

Three hundred forty patients showed positive RT-PCR for COVID-19 infection. Forty-nine of them had a negative first RT-PCR, despite clinical suspicion and high probability chest CT findings. These patients yielded a positive RT-PCR in the second or third undertaking (delayed positive result). One hundred twenty-five patients showed negative RT-PCR results. The sensitivity and specificity of CT to detect novel COVID-19 infection were 92.6% and 67.5% respectively (Fig. 1) (Table 2).

**Table 2:** Diagnostic characteristics of some CT findings in predicting positive RT-PCR

Characteristics	High probability	Intermediate/high probability	Low or more probability	GGO	Crazy paving	Curvilinear opacity	Vascular enlargement
**Sensitivity**	69.7%	81.8%	**92.6%**	85.9%	45.3%	26.5%	29.4%
**Specificity**	**97.6%**	88.9%	67.5%	88.9%	93.7%	96.8%	96.8%
**DA**	77.3%	83.7%	85.8%	**86.7%**	58.4%	45.5%	47.6%
***YI***	67.3%	70.7%	60.1%	**74.8%**	38.9%	23.3%	26.2%
**PPV**	**98.8%**	95.2%	88.5%	95.4%	95.1%	95.7%	96.2%
**NPV**	54.4%	64.4%	**77.3%**	70.0%	38.8%	32.8%	33.7%
**LR+**	29.28	7.36	2.85	7.73	7.13	8.34	9.26
**LR***−*	0.31	0.21	0.11	0.16	0.58	0.76	0.73
**LR**	94.34	35.87	26.12	48.67	12.21	10.98	12.71

*YI* Youden’s index, *DA* diagnostic accuracy, *PPV* positive predictive value, *NPV* negative predictive value, *LR+* positive likelihood ratio, *LR−* negative likelihood ratio, *LR* diagnostic odd ratio

**Fig. 2:**
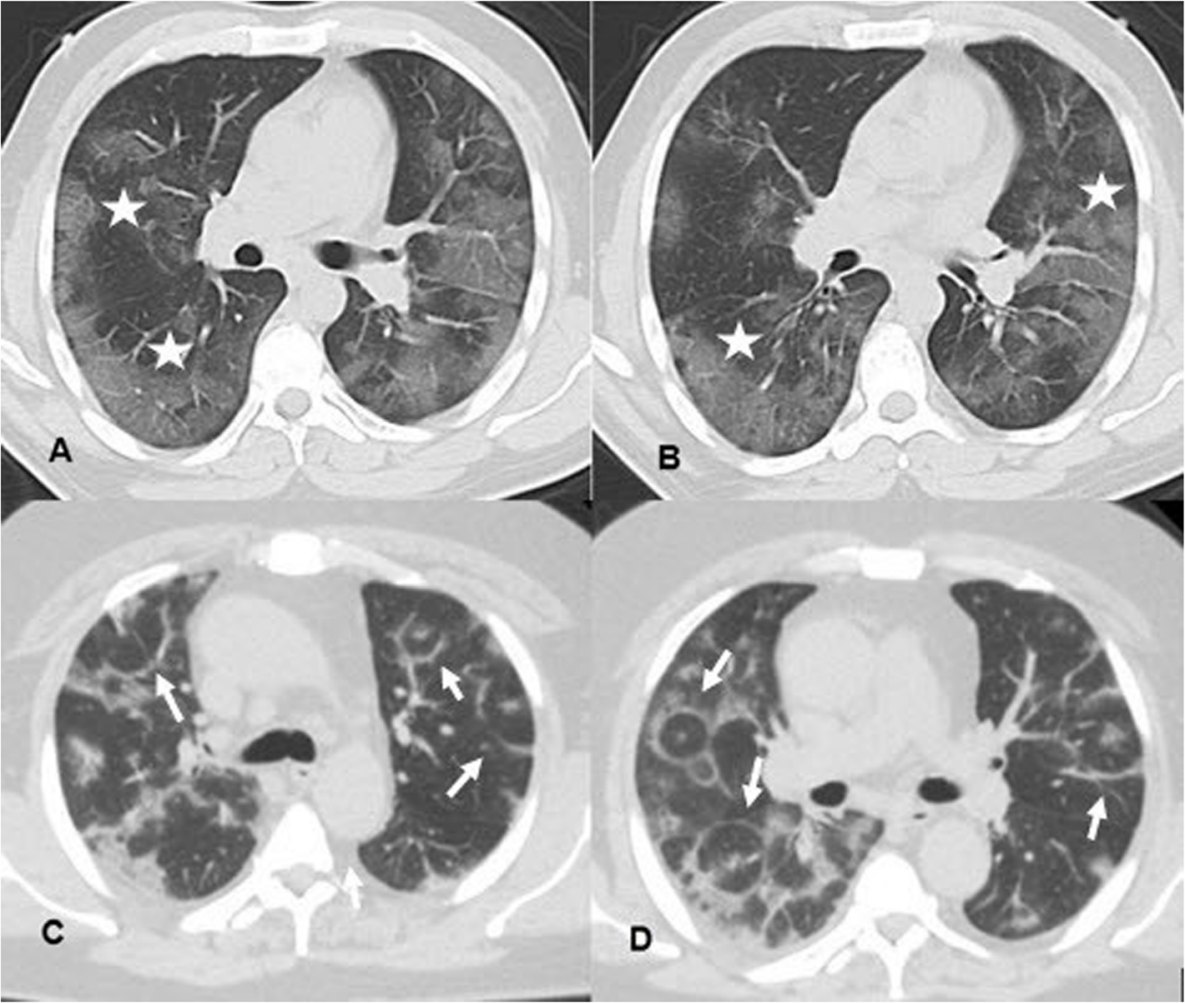
(**A** and **B**) Axial CT images, lung window, of a 38-year-old male who complained of fatigue, fever, and cough of 6 days duration. The images revealed bilateral peripheral multifocal ground-glass opacities with interlobular septal thickening giving the crazy paving appearance (white stars). (**C** and **D**) Axial CT images, lung window, of a 55-year-old male patient who suffered from 10 days of fever, dyspnea, and cough which revealed multiple reverse halo signs (white arrows) and subsegmental consolidation. Both patients reported as high probability cases for COVID-19 following the RSNA recommendations were RT-PCT positive and were hospitalized

**Fig. 3:**
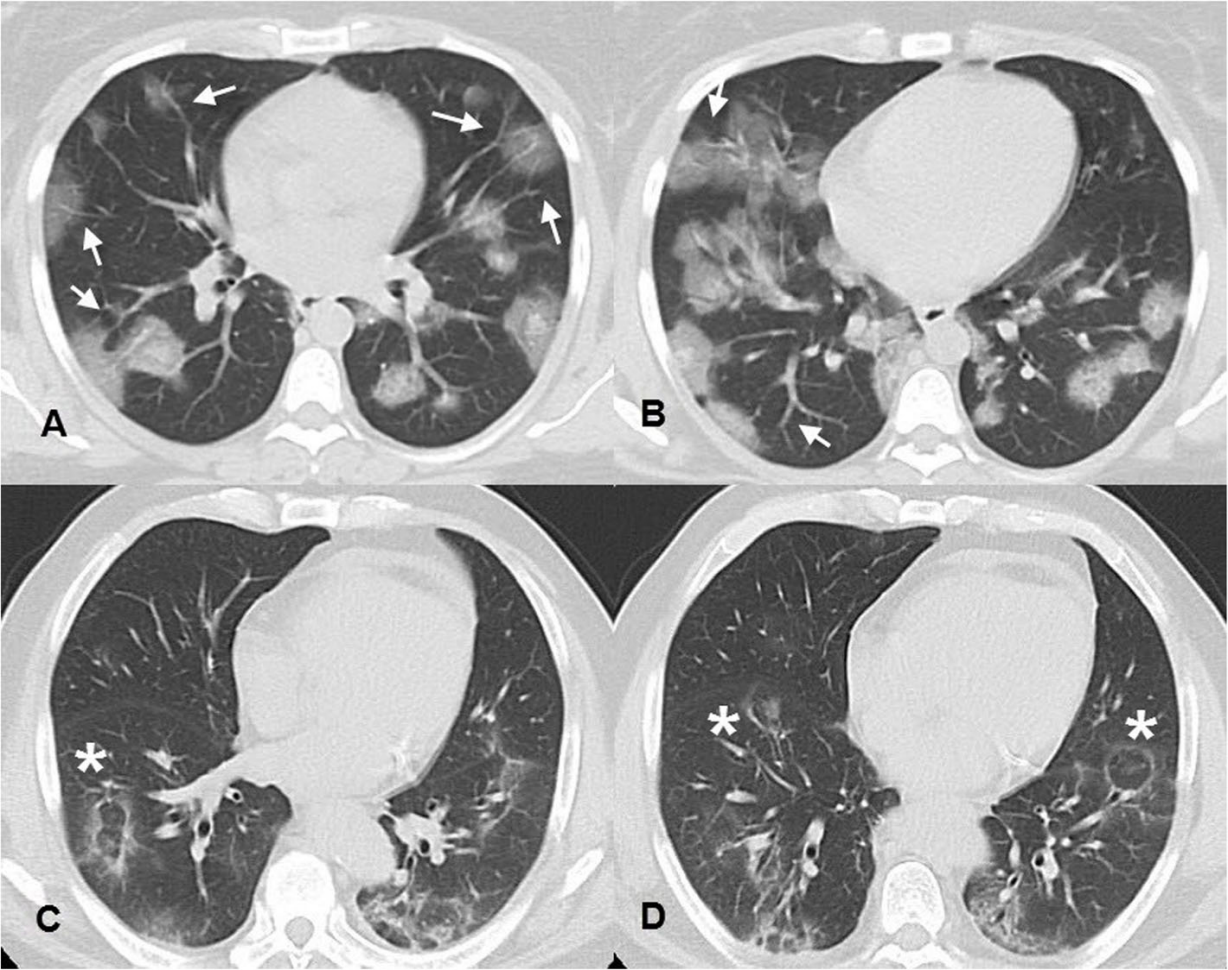
(**A** and **B**) Axial CT images, lung window, of a 29-year-old female patient who attended our hospital complaining of 1-week cough, dyspnea, and fatigue. She tested positive for COVID-19. Her CT was done before the results of the PCR and revealed multiple bilateral scattered rounded ground-glass opacities as well as vascular enlargement (white arrows). (**C** and **D**) Axial CT images, lung window, of a 65-year-old cardiac patient who had fever and dyspnea for 15 days duration and revealed multiple subsegmental consolidations and reverse halo signs (white asterisk). His RT-PCR was positive, and he was hospitalized. Both cases were reported as typical and high probability cases for COVID-19 infection following RSNA recommendations

Reporting following the RSNA recommendations, 240 cases were reported with typical high probability CT findings (Figs. 2 and 3), only three of them had repetitive negative RT-PCR results with an alternative diagnosis was established (2 cases with lupus pneumonitis and 1 case of drug toxicity). Twenty-five positive RT-PCR cases had a negative chest CT when the CT scanning was performed 2-3 days after symptom onset (Fig. 1).

Table 2 revealed a decrease in the CT specificity from the high probability group to the low probability group, as the high probability group showed the highest specificity and PPV.

**Fig. 4:**
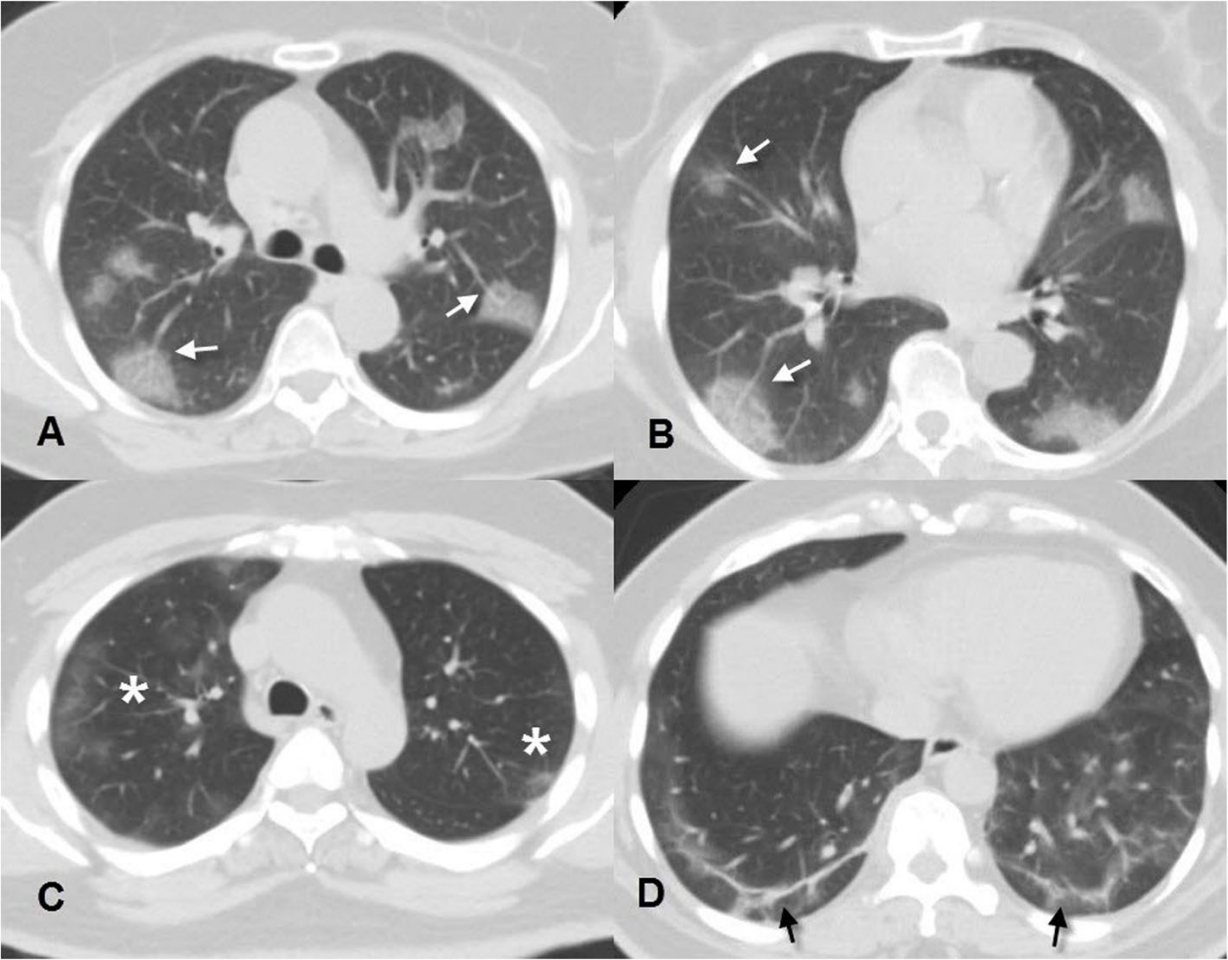
(**A** and **B**) Axial CT images, lung window, of a 60-year-old female who complained of persistent dry cough in the last 6 days. Her images displayed multiple bilateral multifocal subpleural rounded ground-glass attenuation patches with vascular dilatation (white arrows). Her initial RT-PCR was negative, then repeated because of high probability CT findings and was positive. (**C** and **D**) Axial CT images, lung window, of a 40-year-old male patient with fever and dyspnea of 10 days duration, and revealed bilateral upper lobe subpleural patchy ground-glass attenuation areas (white asterisk) as well as bilateral basal subpleural curvilinear opacities (black arrows). He tested positive for COVID-19 and received treatment at home

**Fig. 5:**
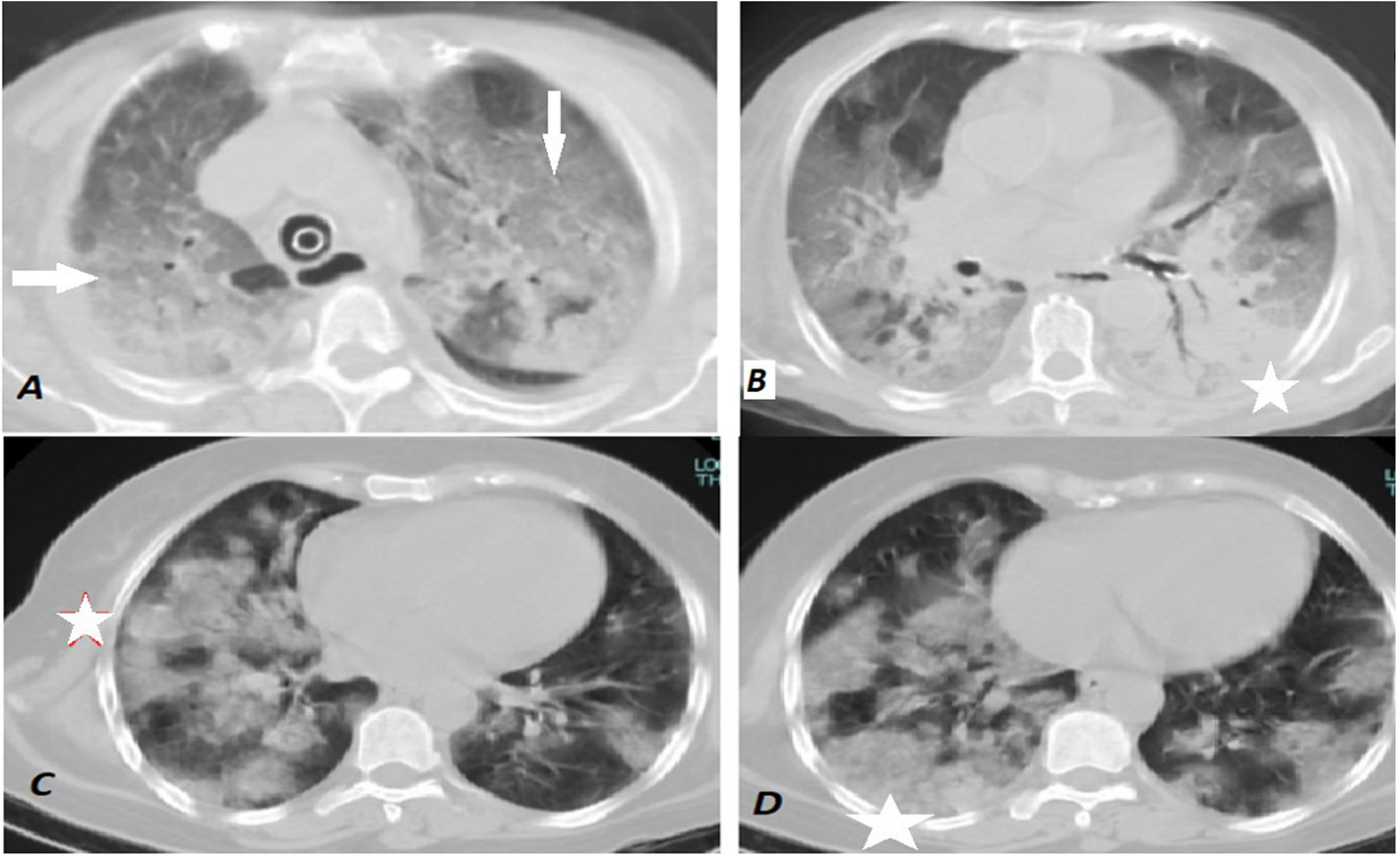
(**A** and **B**) Axial CT images, lung window, of a 63-year-old male who complained of persistent fever, cough, and severe dyspnea in the last 5 days. His images displayed almost bilateral diffuse ground-glass opacities (white arrows) with multiple consolidations mainly involving the left lower lobe (white star). The patient’s CT scan was reported as an intermediate probability for COVID-19 infection and PCR was positive and was admitted to ICU. His CT severity score was 20. (**C** and **D**) Axial CT images, lung window, of a 52-year-old male patient, who suffered from fever and severe dyspnea of 7 days duration and revealed bilateral lower lobar extensive predominant consolidation pattern (predominant consolidation CT phenotype) (white star). His RT-PCR tested positive for COVID-19 and received treatment in ICU. CT severity score was 18

**Fig. 6:**
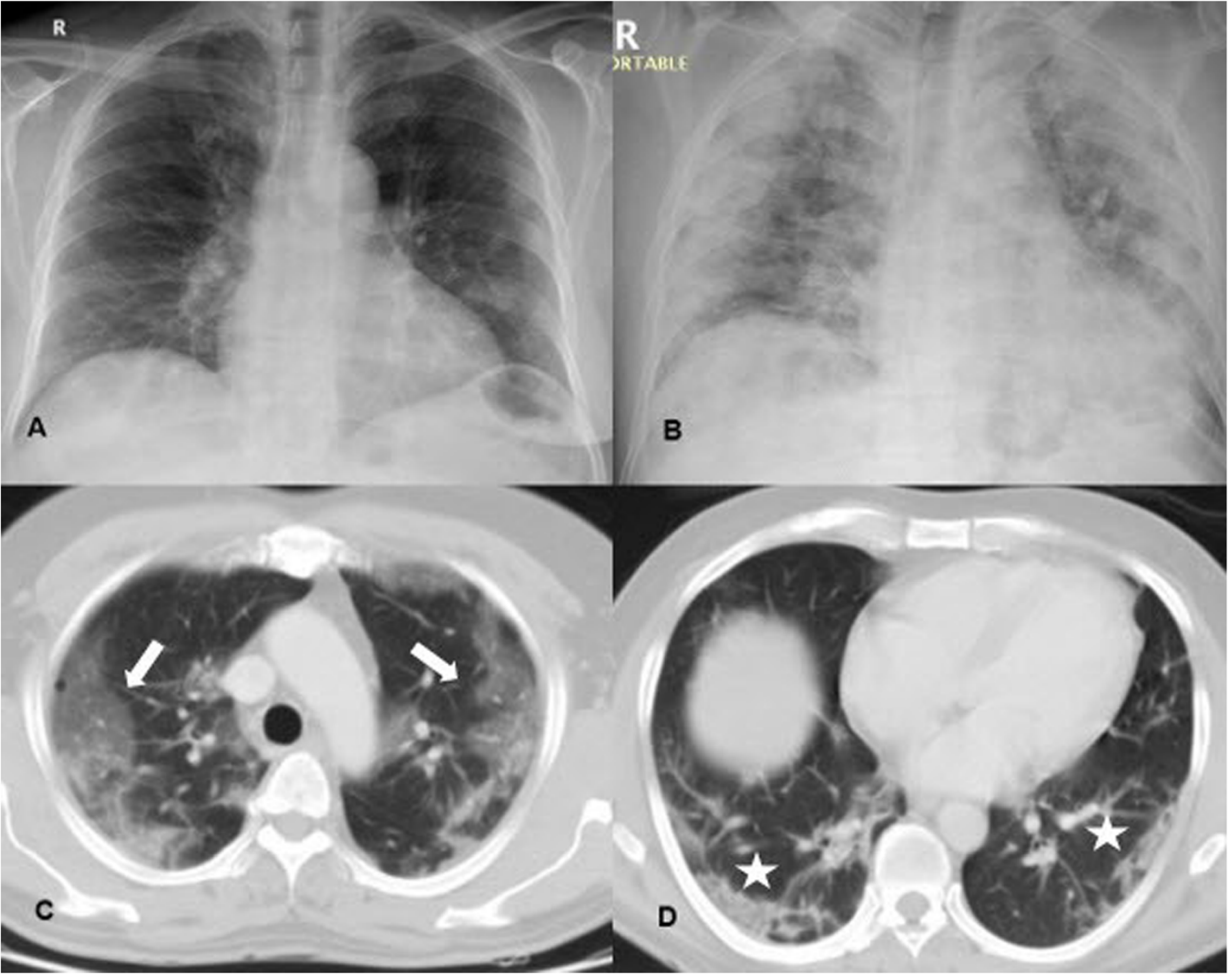
(**A**) Initial normal chest radiograph of a 70-year-old male who was admitted to our hospital with fever, cough, and dyspnea. (**B**) Follow-up chest radiograph after 7 days, the patient’s condition deteriorated, and the radiograph revealed extensive bilateral airspace opacities. (**C** and **D**) Axial CT images, lung window, on the same day of (**B**) which revealed multiple bilateral peripheral subpleural GGO (white arrows) and subsegmental consolidations (white stars) indicating a high probability of COVID-19 and severity score 16. The patient was admitted to the ICU and later on RT-PCR was positive

CT manifestations were noted and recorded during CT interpretation of positive cases illustrated in Table 1 with GGO being the commonest feature, found in 85.9% of positive patients and observed to be predominantly bilateral, multilobar, and peripheral in location (88%, 92.8, and 92.8% respectively) (Fig. 2). The predominant pattern of the GGO was round shape (55.5% of the GGO pattern) (Fig. 4). The second commonest CT feature, noticed among the positive patients, was the crazy-paving pattern, found in 45.3% of positive cases (Fig. 2). Consolidation was found in 28.8% of positive cases. Among them, 74.5% presented with a subsegmental/segmental consolidation pattern. The patients with a predominant consolidation pattern exceeding the GGO were noticed in 15.3% of the patients. So, this study could consider the presence of two CT phenotypes depending on the dominant CT features, one with dominant GGO while the other with dominant consolidations (Figs. 5 and 6).

Consolidation and pleural effusion were significantly distinctive among different management decision groups. Forty-four cases that needed ICU admission showed a consolidation pattern on their chest CT. There was a predominant consolidation pattern compared to the GGO in 66.7% of ICU patients. Also of the 38 positive cases which had a pleural effusion on their CT, 23 of them required ICU admission (Table 3).

**Table 3:** Comparison according to the management decision reflecting the clinical severity

Variables		ICU (*N* = 54)	Hospital (*N* = 70)	Home (*N* = 216)	*P* value
**Mean age (years)**		55.2 ± 15.3 a	51.7 ± 14.5 a	41.7 ± 13.4 b	^ 0.001 *
**Sex**	**Male**	34 (63.0%)	44 (62.9%)	134 (62.0%)	# 0.987
	**Female**	20 (37.0%)	26 (37.1%)	82 (38.0%)	
**Initial negative PCR**		0 (0.0%) a	13 (18.6%) b	36 (16.7%) b	# 0.004 *
**Severity score**		**14.5 ± 6.1 a**	**9.8 ± 5.2 b**	**5.7 ± 3.7 c**	^ < 0.001*
**CT probability**	**High**	33 (61.1%) a	55 (78.6%) a	149 (69.0%) a	# 0.001 *
	**Intermediate**	11 (20.4%) a	4 (5.7%) b	26 (12.0%) ab	
	**Low**	10 (18.5%) a	10 (14.3%) a	17 (7.9%) a	
	**Negative**	0 (0.0%) a	1 (1.4%) a	24 (11.1%) b	
**GGO (ground-glass opacity)**		44 (81.5%)	61 (87.1%)	187 (86.6%)	**#** 0.595
GGO rounded		10 (22.7%) a	25 (41.0%) a	127 (67.9%) b	# < 0.001 *
GGO linear		17 (38.6%) a	17 (27.9%) ab	31 (16.6%) b	# 0.003 *
GGO patchy		10 (22.7%)	8 (13.1%)	31 (16.6%)	**#** 0.426
GGO confluence		5 (11.4%) ab	14 (23.0%) a	20 (10.7%) b	# 0.046 *
GGO diffuse		3 (6.8%) a	0 (0.0%) b	0 (0.0%) b	§ 0.003 *
GGO laterality	Unilateral	3 (6.8%)	5 (8.2%)	27 (14.4%)	**#** 0.222
	Bilateral	41 (93.2%)	56 (91.8%)	160 (85.6%)	
GGO location	Peripheral	36 (81.8%) a	59 (96.7%) b	176 (94.1%) ab	§ 0.014 *
	Central	8 (18.2%)	2 (3.3%)	11 (5.9%)	
GGO lobar affection	Multilobar	43 (97.7%)	58 (95.1%)	170 (90.9%)	**#** 0.249
	Unilobar	1 (2.3%)	3 (4.9%)	17 (9.1%)	
**Crazy paving**		30 (55.6%) ab	42 (60.0%) a	82 (38.0%) b	# 0.001 *
**Vascular enlargement**		17 (31.5%)	18 (25.7%)	65 (30.1%)	**#** 0.733
**Consolidation**		**44 (81.5%) a**	**25 (35.7%) b**	**29 (13.4%) c**	# < 0.001 *
Consolidation type	Lobar	17 (38.6%) a	7 (28.0%) a	1 (3.4%) b	# 0.003 *
	Segmental/subsegmental	27 (61.4%)	18 (72.0%)	28 (96.6%)	
**Consolidation > GG**		**36 (66.7%) a**	**10 (14.3%) b**	**6 (2.8%) c**	# < 0.001 *
**Curvilinear opacity**		12 (22.2%)	20 (28.6%)	58 (26.9%)	**#** 0.713
**Pleural effusion**		**23 (42.6%) a**	**11 (15.7%) b**	**4 (1.9%) c**	# < 0.001 *
**Fibrosis**		4 (7.4%)	3 (4.3%)	10 (4.6%)	§ 0.632
**LN (lymphadenopathy)**		2 (3.7%)	4 (5.7%)	5 (2.3%)	**§** 0.290
**Nodules**		3 (5.6%)	3 (4.3%)	5 (2.3%)	**§** 0.324
**Bronchiectasis**		4 (7.4%) a	0 (0.0%) b	4 (1.9%) b	§ 0.028 *
**Cavitation**		0 (0.0%)	0 (0.0%)	1 (0.5%)	**§** 1.000

^^^Independent t test

^#^Chi-square test

^§^Fisher’s exact test

^*^Significant, homogenous groups had the same symbol (a, b, c) by post hoc Bonferroni test

This study included 39 pediatric patients of which eleven (28.2%) tested positive by RT-PCR. One case (9.1%) presented with peripheral GGO, two cases (18.2%) with predominant central GGO, three cases (27.3%) with consolidation pattern while the remaining 5 (45.4%) showed negative CT findings for pneumonia showcasing the different radiological presentation of COVID-19 in the pediatric age group compared to the adult.

### CT severity scoring

The decision for home isolation was taken for 216 patients, while 120 patients were hospitalized and 55 patients needed ICU admission. A statistically significant relationship was found between the severity score and the decision taken by the Emergency Room team regarding patient management which reflected the severity of the disease (Table 3). Severity score ≥ 14 showed 63% sensitivity, 90.9% specificity, 0.835 AUC for ICU admission (Figs. 5 and 6) while severity score ≤ 10 showed 90.7% sensitivity and 54.8% specificity for home isolation. Consolidation had the highest balanced diagnostic characteristics and NPV among CT findings in predicting ICU admission (Table 4).

**Table 4:** Diagnostic characteristics of some CT findings in predicting patient’s management

Characteristics	ICU			Home		
	Score ≥ 14	Consolidation	Effusion	Score ≤ 10	Consolidation	Effusion
**AUC**	0.835			0.793		
**SE**	0.032			0.026		
**95% CI**	0.772-0.898			0.742-0.845		
**Sensitivity**	63.0%	81.5%	42.6%	90.7%	86.6%	98.1%
**Specificity**	90.9%	81.1%	94.8%	54.8%	55.6%	27.4%
**DA**	86.5%	81.2%	86.5%	77.6%	75.3%	72.4%
***YI***	53.9%	**62.6%**	37.3%	**45.6%**	42.2%	25.6%
**PPV**	56.7%	44.9%	60.5%	77.8%	77.3%	70.2%
**NPV**	92.9%	**95.9%**	89.7%	77.3%	70.4%	89.5%
**LR+**	6.93	4.32	8.12	2.01	1.95	1.35
**LR***−*	0.41	0.23	0.61	0.17	0.24	0.07
**LR**	17.00	18.90	13.40	11.90	8.09	20.02

*AUC* area under the curve, *SE* standard error, *CI* confidence interval, *YI* Youden’s index, *DA* diagnostic accuracy, *PPV* positive predictive value, *NPV* negative predictive value, *LR+* positive likelihood ratio, *LR−* negative likelihood ratio, *LR* diagnostic odd ratio

There was a statistically significant incidence of consolidation between the old age group (≥ 60) and the predominant consolidation pattern in the extreme of ages (Table 5). The old age group (≥ 60) shows a higher incidence of ICU admission and hospitalization.

**Table 5:** Comparison of the age of the study population with the consolidation pattern and CT severity score

Variables		≤ 18.0 (*N* = 39)	19.0–59.0 (*N* = 340)	≥ 60.0 (*N* = 87)	*P* value
**Consolidation**		13 (33.3%) ab	76 (22.4%) a	39 (44.8%) b	# < 0.001 *
**Consolidation > GG**⌂		12 (30.8%) a	38 (11.2%) b	27 (31.0%) a	# < 0.001 *
**Consolidation site**	**Lobar**	8 (61.5%) a	17 (22.4%) b	17 (43.6%) ab	# 0.005 *
	**Segmental/subsegmental**	5 (38.5%)	59 (77.6%)	22 (56.4%)	
**Clinical severity**	**ICU**	2 (18.2%) ab	30 (11.3%) b	22 (34.9%) a	# < 0.001 *
	**Hospital**	1 (9.1%) ab	47 (17.7%) b	22 (34.9%) a	
	**Home**	8 (72.7%) a	189 (71.1%) a	19 (30.2%) b	

^#^Chi square test

^*^Significant

^⌂^From column total

Homogenous groups had the same symbol (a, b, c) by post hoc Bonferroni test

## Discussion

Due to the tremendous rise in the number of COVID-19 patients received by our institute and the shortage or sometimes inconsistency of RT-PCR testing, a search for simple and rapid tools to support the clinical and laboratory findings was mandatory.

In this article, the use of chest CT to help diagnose and facilitate clinical decision-making in suspected COVID-19 patients was discussed. Following the Fleischner society recommendation, one CT scanner was preserved for scanning COVID patients following the instruction of the infection control to avoid infection transmission while other scanners were used for regular medical service [16].

The sensitivity of CT in COVID-19 compared to RT-PCR in the present study was 92.6%. Findings resembled the study done by Wang Y et al. [13] who concluded a CT sensitivity of 84%. However, this was lower than other studies which concluded CT sensitivity of 97-98% [11, 17, 18]. The possible explanation for this difference is that 7.4% of our positive cases had normal CT which was performed too early (2-3 days after the onset of symptoms). Bernheim A et al. [19] found 50% of patients had a normal CT at 0-2 days after onset of symptoms.

According to RSNA recommendations, high probability CT criteria were found in 69.7% of positive cases, showed the highest specificity (97.6%) and PPV (98.8%). These results were similar to Fang et al. [18] who reported 72% of the patients with typical CT findings. Also, 7.4% of the positive cases showed a negative CT pattern which indicated that negative CT should not be used to exclude COVID-19 infection and was consistent with ACR recommendations [20]. The specificity of the CT probability, following the RSNA recommendations, was higher in the high probability group (97.6%) and decreased gradually in the low probability group (67.5%) which denoted an increase in the number of true positive cases among the CT high probability patients. A finding that was similar to Jaegere et al. [20].

CT patterns of COVID-19 patients, GGO was the predominant pattern in the current study and was found in 85.9% of positive patients with predominant round morphology (55.5%). Bilateral, peripheral, and multilobar distribution of the GGO was predominant. This was similar to Salehi et al. [21] who studied 919 patients and found GGO in 88% of cases. Ojha V et al. [22] reviewed 45 studies including 4410 adult patients. They found isolated GGO in 50.2% and combined with consolidation in 44.4%. Other studies found a higher incidence of the GGO in positive cases reaching 98-100% [11, 23]. Bilateral, peripheral, and multilobar distribution of the GGO was found to be the most specific feature for diagnosis of COVID-19 infection and had the highest incidence [10, 23, 24].

The second most common CT manifestation was the crazy-paving pattern, which was found in 45.3%. This was followed by a vascular dilatation sign that was detected in 29.4% of the positive cases. Ojha V et al. [22] found crazy-paving in 19.5% of the positive cases and described vascular dilatation in 64% of cases. Li et al. [23] found a crazy-paving pattern in 36%. Bai et al. [25] described vascular enlargement signs in 59% of their COVID-19 patients.

Consolidation is a sequel of replacement of alveolar air by cellular fibromyxoid exudate which might be considered as a sign of disease progression and severity [26]. In patients with novel COVID-19 infection, the incidence of consolidation reported in the CT studies ranges from 2-64% [8]. In the current study, it was present in 28.8% of positive cases. It was noticed that the incidence of consolidation between ICU patients was high (81.5%). The consolidation pattern was the dominant pattern in 66.7% of the ICU patients. If we can consider the presence of two CT phenotypes for the COVID-19 infection as found in the result, one with predominant GGO while the other with predominant consolidation. The latter one with a predominant consolidation pattern is the more risky phenotype carrying a worse prognosis.

Salehi et al. [21] and Chung et al. [27] found consolidation in 31% and 29% of the cases which are similar to the result of the current study. Also, Ojha V et al. [22] found consolidation in 24.2% of the positive cases and also noticed the increasing incidence of the consolidation between the older age group and patients who had severe pneumonia.

Additionally, Song et al. [28] found that consolidations were more in patients with delayed CT after the onset of their symptoms and in patients older than 50 years.

Subpleural curvilinear lines occurred in COVID-19 patients likely due to pulmonary edema or early fibrosis [8]. In the present study, 28.6% of positive cases showed curvilinear pulmonary parenchymal lines on their chest CT. This was similar to Ojha et al. [22] who reported a 25% incidence of subpleural lines. Li et al. [23] and Wu et al. [29] found curvilinear lines in 20% of cases.

Pleural effusion was detected in 11.2% of positive cases which was similar to Li et al. [23] and Song et al. [28] who found pleural effusion in 8% of their study population. Pleural effusion was found in 42.6% of ICU patients, so it might be considered as a sign of disease progression and the need for ICU admission [22, 23, 30].

Airway changes in the form of bronchiectasis occurred due to extensive inflammatory damage with fibrous tissue formation and subsequent traction bronchiectasis. Some authors have considered it a sign of severity [8]. Bronchiectasis was found in 2.4% of positive cases and this was lower than the results reported by Ojha et al. [22] and Shi et al. [30] which were 18% and 11% respectively.

Fibrosis occurred likely due to the healing process. It is not clear whether it was a good or a poor prognostic sign [31, 32]. The present study recorded only 5% of the positive patients with pulmonary parenchymal fibrosis on their chest CT scans. Ojha et al. [22] found a higher incidence of fibrosis (17.4%).

Lymph nodes, nodules, and cavitation were the least common CT findings between the current study sample population and these were similar to most of the researches [8].

Concerning the CT severity of COVID-19, the current study showed a significant correlation between the CT severity and the patients’ management decision. The cut-off value of ≥ 14 preferred ICU admission while the cut-off value of ≤ 10 favored home isolation.

Yang R et al. [33] studied the CT severity score in 102 positive patients. They correlated the score with the clinical severity and found a higher CT severity score in severe COVID-19 infection when compared with the mild cases. They calculated a cut-off value of 19.5 out of 40 with 83.3% sensitivity and 94% specificity.

In the present study, sufficient data were not available on COVID-19 infection in pediatric patients. Only 39 symptomatic pediatric patients were examined in this study and 11 (3.2%) had positive RT-PCR. The low incidence of COVID-19 infection among the pediatric age group in our study was almost similar to most of the available studies about the COVID-19 infection in pediatric population (1.2-5.2%) [34, 35]. Only one patient showed typical and high probability CT findings, while the others presented with negative, low, and intermediate CT probabilities (5, 3, and 2 patients respectively). The chest CT findings of COVID-19 infection among pediatric patients were different from those usually found in adults. These were concluded by others [34, 35].

### Limitation

Owing to the pandemic status and the increasing number of patients, it was not possible to collect full laboratory data about each of the patients which of course was of additive value if compared to the CT scoring. So, we depend on the clinical suspicion as well as we used the decision taken for the patient’s management as a reflection of their clinical condition. Also, during image interpretation any difference in interpretation was resolved by consensus to avoid any inter-observer disagreement as this was not the scope in this study which may make a some bias, so further studies is recommended to study the inter and intra-observer agreement degrees.

## Conclusion

CT can be used as a rapid and sensitive tool aiding in the rapid diagnosis and management of COVID-19 infection. It can help in early isolation and limit the dissemination of the disease. The RSNA recommendations in reporting suspected COVID-19 cases were valuable in the current study keeping given that a negative CT result can be found in the early course of the disease. It is recommended to perform a CT chest at least 5 days after the onset of symptoms to avoid false-negative results. The two CT phenotypes for COVID-19 infection were predominant/purely GGO and the predominant consolidation pattern. The latter is a red flag for severe infection and ICU admission. CT severity score reflecting the disease distribution and reflecting the degree of pulmonary involvement and can be used in the prediction of the disease outcome and need for ICU.

## Data Availability

Available on request with the corresponding author.

## Abbreviations

COVID-19: Coronavirus disease 2019
ICU: Intensive care unit
RSNA: Radiological Society of North America
ACR: American College of Radiology
ER: Emergency room
GGO: Ground-glass opacity
CT: Computed tomography
RT-PCR: Real-time reverse transcription-polymerase chain reaction
NCIP: Novel COVID-19-infected pneumonia
